# PDE3 Inhibition Reduces Epithelial Mast Cell Numbers in Allergic Airway Inflammation and Attenuates Degranulation of Basophils and Mast Cells

**DOI:** 10.3389/fphar.2020.00470

**Published:** 2020-05-01

**Authors:** Jan Beute, Keerthana Ganesh, Hedwika Nastiti, Robin Hoogenboom, Vivica Bos, Jelle Folkerts, Marco W. J. Schreurs, Steve Hockman, Rudi W. Hendriks, Alex KleinJan

**Affiliations:** ^1^Department of Pulmonary Medicine, Erasmus MC, Rotterdam, Netherlands; ^2^Department of Immunology, Erasmus MC, Rotterdam, Netherlands; ^3^Flow Cytometry Core of the National Heart, Lung, and Blood Institute, NIH, Bethesda, MD, United States

**Keywords:** allergic airway, animal model, asthma, enoximone, house dust mite, inflammation, PDE3, PDE4

## Abstract

Epithelial mast cells are generally present in the airways of patients with allergic asthma that are inadequately controlled. Airway mast cells (MCs) are critically involved in allergic airway inflammation and contribute directly to the main symptoms of allergic patients. Phosphodiesterase 3 (PDE3) tailors signaling of cyclic adenosine monophosphate (cAMP) and cyclic guanosine monophosphate (cGMP), which are critical intracellular second messenger molecules in various signaling pathways. This paper investigates the pathophysiological role and disease-modifying effects of PDE3 in mouse bone marrow-derived MCs (bmMCs), human LAD2- and HMC1 mast cell lines, human blood basophils, and peripheral blood-derived primary human MCs (HuMCs). In a chronic house dust mite (HDM)-driven allergic airway inflammation mouse model, we observed that PDE3 deficiency or PDE3 inhibition (PDE3i) therapy reduced the numbers of epithelial MCs, when compared to control mice. Mouse bone marrow-derived MCs (bmMCs) and the human HMC1 and LAD2 cell lines predominantly expressed PDE3B and PDE4A. BmMCs from *Pde3^−/−^* mice showed reduced loss of the degranulation marker CD107b compared with wild-type BmMCs, when stimulated in an immunoglobulin E (IgE)-dependent manner. Following both IgE-mediated and substance P-mediated activation, PDE3i-pretreated basophils, LAD2 cells, and HuMCs, showed less degranulation than diluent controls, as measured by surface CD63 expression. MCs lacking PDE3 or treated with the PDE3i enoximone exhibited a lower calcium flux upon stimulation with ionomycine. In conclusion PDE3 plays a critical role in basophil and mast cell degranulation and therefore its inhibition may be a treatment option in allergic disease.

## Introduction

Mast cells (MCs) play a key role in human allergic airway inflammation. The observation of bronchial epithelial MCs in asthmatic patients (Braunstahl et al., 2003) is an indication that the presence and location of MCs are important. Mast cell and basophil dynamics were also observed in the nasal mucosa of allergic rhinitis patients (KleinJan et al., 2000). The findings of elevated numbers of MCs and increased serum tryptase levels, suggesting MC activation, implicate MCs in various lung diseases, including idiopathic pulmonary fibrosis and sarcoidosis (Bargagli et al., 2009; Wygrecka et al., 2013). It has been reported that the presence of pulmonary neuroendocrine cells amplify allergic asthma responses by the release of the neuropeptides calcitonin gene-related peptide (CGRP) and substance P (SP), both of which can act as MC stimulators (Sui et al., 2018).

Localization of MCs is an important pathophysiological feature; in healthy controls they are absent in the epithelium but present in submucosal airways. The presence of MCs in the epithelium or in close proximity to smooth muscle airway cells (Brightling et al., 2002; Bradding et al., 2006; Bradding and Brightling, 2007) contributes to smooth muscle hyperplasia *via* TGFβ and β-tryptase (Woodman et al., 2008). In uncontrolled allergic asthma patients the total number of MCs and MC_TC_ (MC containing tryptase and chymase) in the alveolar parenchyma was found to correlate negatively with FEV1% predicted (Andersson et al., 2011; Andersson et al., 2018). In these patients the numbers of mast cells expressing FcεR1 and TGFβ are increased. These findings indicate the connection between disease and parenchymal MCs in uncontrolled asthmatics. In addition, the amount of collagen deposition correlates with the number of MCs in the parenchyma (Andersson et al., 2011). *In vivo* mast cell studies are hampered by the fact that staining for serine proteases is not always easy to interpret because MCs degranulate during allergen challenge; the number of serine protease-positive cells drops, because degranulated cells are not positive anymore (Balzar et al., 2011). Basophil and MC accumulation occurs in the airways after allergen inhalation and/or challenges of allergic patients (Gauvreau et al., 2000; KleinJan et al., 2000; Braunstahl et al., 2003), and in fatal asthma (Perskvist and Edston, 2007; Woodman et al., 2008; Yu et al., 2011). In allergy, mast cell and basophil degranulation is initiated during the early-phase reaction and continues to the late-phase reaction (Togias et al., 1988; Fokkens et al., 1992; de Graaf-in't Veld et al., 1997; KleinJan et al., 2000).

MC activation by immunoglobulin E (IgE)-dependent (i.e., “allergic”) or other mechanisms release a diverse spectrum of mediators that induce local effects on blood vessels, nerves, mucous glands, epithelial cells, airway smooth-muscle cells, and immune cells (Bradding et al., 2006). Analyses in chronic asthma mouse models indicated that MCs can contribute to the establishment of chronic eosinophilic airway inflammation (Yu et al., 2011). They also contribute to features of tissue remodeling that resemble those observed in asthma patients, including increased numbers of mucus-secreting goblet cells in the airway epithelium and increased deposition of interstitial collagen (Yu et al., 2011; Li et al., 2019).

In the context of allergic airway inflammation and asthma, phosphodiesterase 3 (PDE3) and PDE4 are widely expressed in airway cells, including epithelial and endothelial cells, smooth muscle cells, and inflammatory cells (Beavo, 1995; Blease et al., 1998; Wright et al., 1998; Myou et al., 1999; Souness et al., 2000; Boswell-Smith et al., 2006). Whereas both PDE3 and PDE4 are cyclic adenosine monophosphate (cAMP)-degrading enzymes, only PDE3 degrades cyclic guanosine monophosphate (cGMP) as well. PDE4 is an asthma-susceptibility gene (Himes et al., 2009). PDE inhibition (PDEi) appears to act effectively on immune cells, as well as on endothelial cells, epithelial cells, and airway smooth muscle cells (KleinJan, 2016). PDE3 deficiency or pharmacological inhibition of PDE3 activity was shown to reduce allergic airway inflammation and to improve airway mucosal barrier function in allergic airway models (KleinJan, 2016). However, the role of PDE3 and the effects of PDE3i on MC and basophil degranulation remained unknown.

This paper investigates the pathophysiological role of PDE3 in MCs and basophils *via in vitro* studies. Based on the observations that i) PDE3 deficiency or PDE3i reduces allergic airway inflammation (Beute et al., 2018) and that ii) epithelial MCs are induced in a chronic house dust mite (HDM)-driven asthma model (Beute et al., 2018; Li et al., 2019), III) and that elevated cAMP levels have an inhibitory effect on exocytosis (Alm, 1984) we hypothesize that PDE3 activity is important for mast cell function. To determine how PDE3 deficiency or PDE3i affects mast cell function, we analyzed mast cells in a chronic HDM asthma model and investigated *in vitro* degranulation. We observed that in cell cultures PDE3 inhibitors prevented and/or attenuated stimulation-induced degranulation of bone marrow-derived mouse mast cells, human peripheral blood mononuclear cell (PBMC) basophils, LAD2 mast cell lines, and peripheral blood-derived human mast cells.

## Results

### The Presence of Epithelial Mast Cells Is a Sign of Chronic Allergic Airway Inflammation

**Figure 1:**
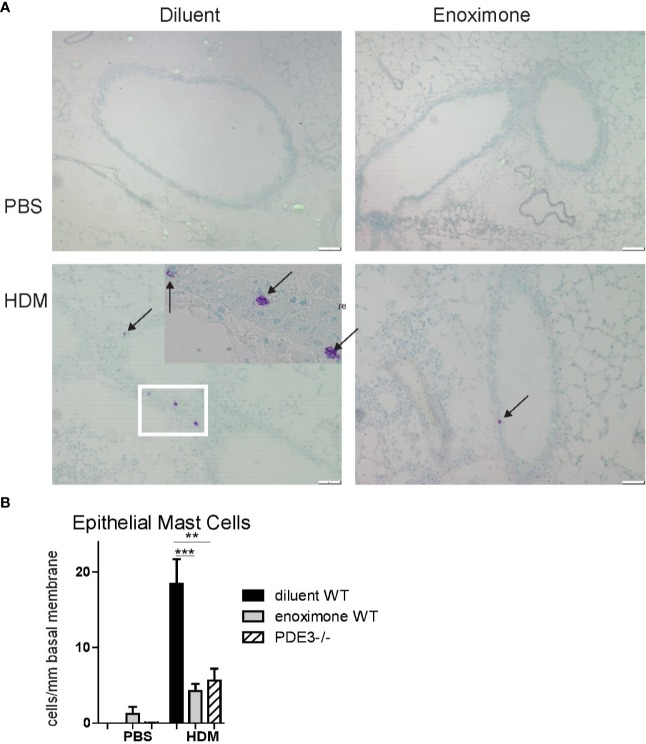
Mast cell recruitment is impaired when PDE3 is targeted. **(A)** Representative photographs of toluidine blue (arrows pointed to mast cells) stained lung section obtained from phosphate buffered saline (PBS) control mice and house dust mite (HDM) driven asthmatic mice. **(B)** Number of epithelial mast cells (MCs) per mm length basal membrane. A Kruskal-Wallis test for multiple comparisons was used, followed by a Mann-Whitney U test. Error bars show mean values ± SEM (n=3 for PBS, n=6 for HDM groups; **P < 0.01; ***P < 0.005). Data are representative for three independent experiments.

Evaluation of PDE mRNA transcripts indicated the expression of PDE3B, PDE4A, PDE4B, and PDE4D in mouse bone marrow-derived MCs. Hardly any PDE3A expression was detected and no PDE4C (**Supplementary Figure 1**). In earlier studies the presence of airway epithelial MCs was demonstrated in allergic rhinitis and allergic asthma patients (KleinJan et al., 2000; Braunstahl et al., 2003) as well as in chronic HDM-driven asthma models (Li et al., 2019). MCs contribute to the development of multiple features of asthma pathophysiology, both in an IgE-dependent way and in an IgE-independent neurogenic-driven way (Devos et al., 2016; Sibilano et al., 2016). By toluidine blue staining we investigated the presence of MCs in airway epithelium samples in a chronic HDM-driven asthma model. No epithelial MCs were present in control phosphate buffered saline (PBS) mice, but they did appear following HDM treatment. Degranulated—and therefore activated—MCs were seen in HDM-exposed placebo-treated wild-type (WT) mice (**Figure 1A** and insert). Mice lacking PDE3 (*Pde3^−/−^*) or mice that received topical (**Supplementary Figure 2**) PDE3i therapy showed lower (p < 0.01 and p < 0.005 resp.) epithelial MC numbers and those MCs present were in a more granulated stage (**Figure 1B**). Other airway structures, including parenchyma and smooth muscle areas, were negative for mast cells. In conclusion: epithelial mast cell numbers were lower in a HDM-driven allergic airway inflammation mouse model when PDE3 is lacking or when mice were treated with PDE3 inhibitors.

### PDE3 Plays a Pathophysiological Role in Mast Cell Degranulation

**Figure 2:**
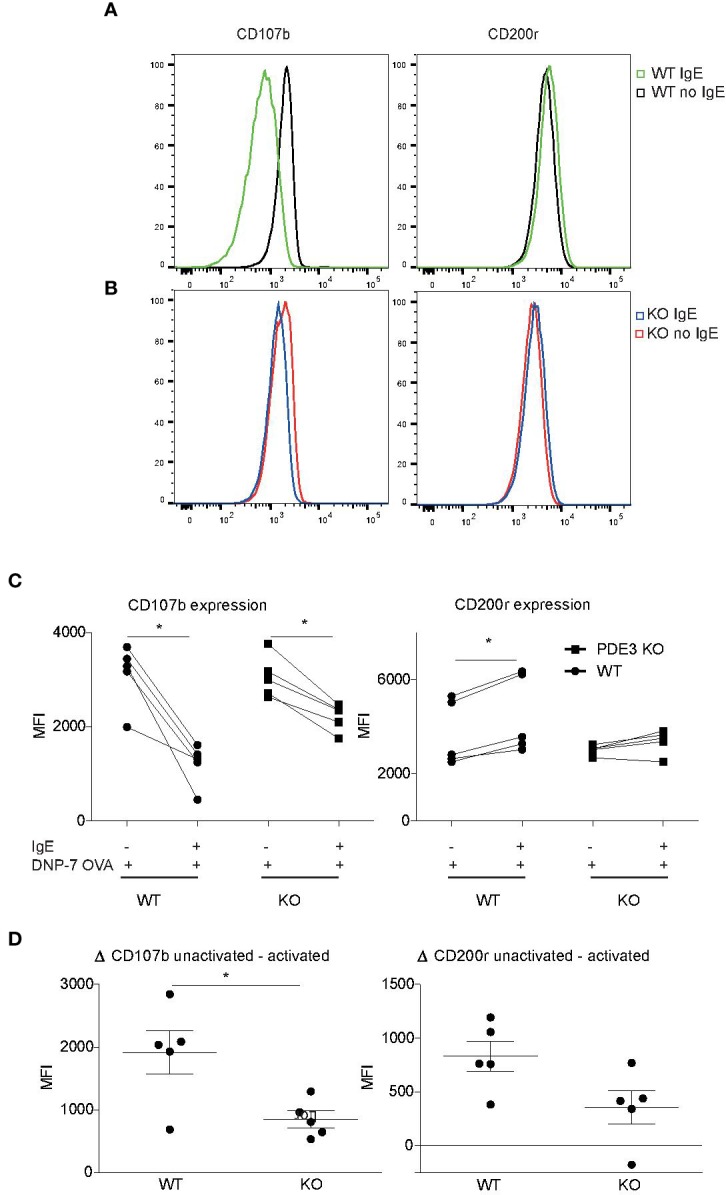
Mast cell activation is impaired when PDE3 is targeted. **(A, B)** Bone marrow-derived mast cells (MCs) obtained form *Pde3^−/−^* mice and wild-type (WT) control mice were sensitized with DNP-specific immunoglobulin E (IgE) or phosphate buffered saline (PBS), followed by stimulation with DNP-7 OVA. The mast cell activation markers CD107b and CD200r were evaluated and data are shown as histogram overlays. **(C)** Dot plots with median fluorescence intensity (MFI) values indicating reduction in CD107b and induction of CD200r upon DNP-7 OVA stimulation for WT and *Pde3^−/−^* mast cells. **(D)** ΔMFI for the indicated markers in MCs from WT and *Pde3^−/−^* mice (KO). A Wilcox signed test and a Mann-Whitney U test was used for statistical evaluation. *P < 0.05. Plots represent (n=5) of one of two independent experiments. MFI, median fluorescence intensity.

*In vitro*-cultured mouse bone marrow-derived MCs (bmMCs) were examined to investigate the role of PDE3 in MC degranulation, using changes in CD107b and CD200r expression as markers. Mast cell expression of surface CD200r was shown to be increased after both IgE- and non-IgE-mediated activation (Larson and Mitre, 2012). Overnight 2,4-dinitrophenyl (DNP)-specific IgE-sensitized MCs and PBS control MCs were stimulated with 10 ng/ml DNP-7-OVA for 30 min. IgE-receptor cross-linking *via* DNP-7-OVA occurred only in DNP-specific IgE-loaded MCs and showed lower levels of CD107b or higher levels of CD200r when compared to controls (**Figures 2A, C**). *Pde3^−/−^* MCs were less activated upon FcεR cross-linking when measured with MCs degranulation markers CD107b and CD200r (**Figures 2A–D**). Unstimulated WT and *PDE3^−/−^* MCs showed no significant differences in median fluorescence intensity (MFI) for CD107 or CD200r. In conclusion: mouse MCs lacking *PDE3* were less activated upon FcεR cross-linking.

### Clinically Available PDE3 Inhibitors Inhibit Basophil Degranulation

**Figure 3:**
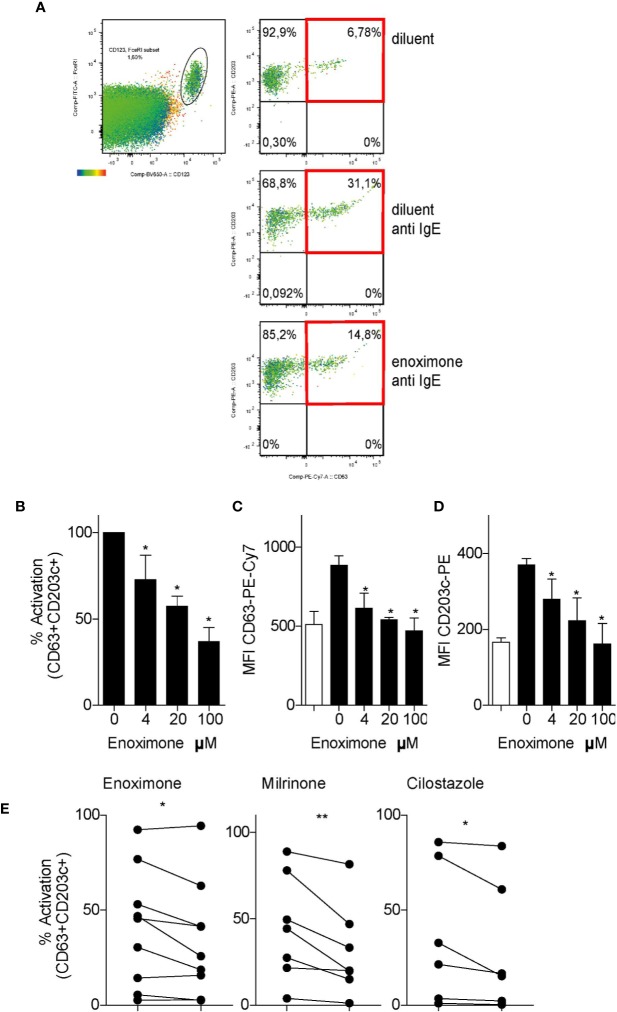
PDE3 inhibitors reduce FcεR-mediated basophil activation. **(A)** Flow cytometry (FACS) plot of CD63/CD203 profiles of the CD123^+^FcεR^+^ basophil cell fractions from PBMCs that were treated with enoximone or diluent and stimulated with phosphate buffered saline (PBS) or anti-immunoglobulin E (IgE), as indicated. **(B–D)** Dose-response to enoximone for proportions of CD63^+^CD203^+^ activated cells within the fraction of CD123^+^FcεR^+^ PBMC **(B)**, MFI values for CD63 **(C)**, and MFI values for CD203c **(D)**. Values are mean ± S.E.M; n=3. **(E)** Capacity of the PDE3i enoximone, milrinone and cilostazol to inhibit activation of basophils, shown as the proportions of CD63^+^CD203^+^ activated cells within the fraction of CD123^+^FcεR^+^ PBMC. A Wilcox signed test and a Mann-Whitney U test was used. *P < 0.05; **P < 0.01. Data are shown of one representative experiment from three independent experiments. MFI, median fluorescence intensity.

To study the effect of PDE3i on the degranulation of human basophils in the PBMCs fraction, a modified basophil-degranulation test was performed. Basophils were defined as FcϵRI^+^HLA-DR^−^CD123^+^ cells, and further analyzed for CD63 and CD203c^+^ expression as degranulation markers. Because CD63 expression is a marker for MC activation (Cop et al., 2018; Taracanova et al., 2018), we determined that, when basophils were activated by FcεR cross-linking, the population of CD63^+^CD203c^+^ cells increased from ~7 to ~31% (**Figure 3A**). As quantified by the proportions of CD63^+^CD203c^+^ cells, anti-IgE-activated basophils showed a diminished degranulation status, when pre-treated with enoximone in a dose-dependent manner (**Figures 3B–D**). Attenuation of these degranulation markers was achieved even at a low dose of 4 µM enoximone. Other PDE3i (milrinone 100 µM and cilostazol 100 µM) were able to impair basophil degranulation as well (**Figure 3E**).

### Mast Cell Line Degranulation Is Impaired When PDE3 Is Targeted

**Figure 4:**
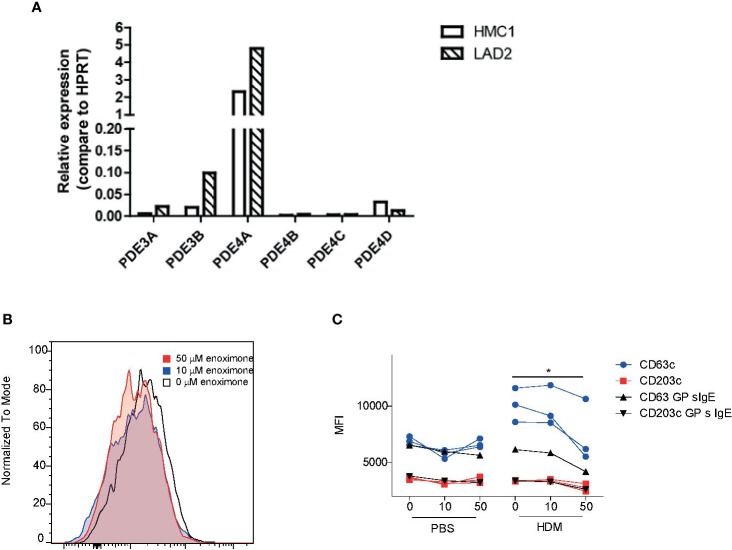
Effect of enoximone on FcεR-mediated activation of human mast cell lines **(A)** Expression of PDE3 and PDE4 isoforms in the human mast cell lines indicated, as determined by real-time (RT)-PCR. **(B, C)** Activation of HDM-stimulated serum immunoglobulin E (IgE) passively sensitized LAD2 cells, as determined by flow cytometry. Data are shown as histograms overlays for CD63 **(B)** and median fluorescence intensity (MFI) (CD63 and CD203c) of flow cytometry (FACS) measurements of enoximone (µM) pretreated and HDM (10 ng/ml) stimulated passive sensitized (HDM specific serum IgE) LAD2 cells **(C)**. A Wilcox signed test was used; *P < 0.05. Data are shown from one out of two independent experiments. MFI, median fluorescence intensity; HDM, house dust mite.

HMC1 and LAD2 mast cell lines express PDE3 and PDE4 transcripts (**Figure 4A**). Attenuation of surface degranulation markers in LAD2 cells was observed only if we treated the cells with higher doses of enoximone (**Supplementary Figure 3A**). For these experiments, sera obtained from HDM mono-allergic (68-121 KU/l) or grass pollen (GP) mono-allergic (123-211 KU/l) donors were used. Overnight passively sensitized LAD2 cells with sera (controls: 20% serum or PBS) were stimulated with PBS or HDM. When the cells were pretreated with enoximone in a dose-dependent manner, followed by HDM stimulation (1 ng/ml), the CD63- and CD203c-expression levels on the surface of LAD2 (**Figure 4B**) cells were lower. Control LAD2 cells sensitized with serum IgE obtained from isolated GP-allergic subjects showed no degranulation upon HDM stimulation (**Figure 4C**). The marker CD203c presented less sensitive than CD63 in MCs (**Figure 4C**).

### PDE3 and Cyclic Adenosine Monophosphate Both Contribute to the Regulation of Mast Cell Degranulation

PDE3 inhibitors increase the intracellular cAMP and/or cGMP concentrations. The effects of cAMP- or cGMP-analogue treatment on the expression of CD63 on LAD2 cells were evaluated (**Supplementary Figures 3A–D**) following 1 μM SP stimulation. LAD2 cells showed reduced CD63 expression when pretreated with enoximone (1,000 µM) followed by SP stimulation. SP stimulation of LAD2 cells pretreated with a high concentration of cAMP (1 mM) analogue was able to prevent CD63 expression altogether (**Supplementary Figure 3B**). Treatment using cGMP (1 mM) analogue and stimulation with SP showed no effect on CD63 expression (**Supplementary Figure 3C**). One of the downstream targets of the cAMP pathway is protein kinase A (PKA). To investigate the molecular mechanism of PDE3 inhibition in LAD2 cells, we stimulated LAD2 cells that were pretreated with PKA inhibitor (Rp)-8-Br-cAMP (100 µM) with SP, and observed a significant reduction of CD63 expression. This suggests that the LAD2-cell modulation of CD63 expression is regulated, at least in part, *via* the cAMP-PKA pathway.

Next, the Ca^2+^ flux in MCs lacking PDE3 or treated with PDE3i was investigated. Antigenic stimulation and neurogenic stimulation were bypassed through the use of ionomycin, a calcium ionophore that opens all Ca^2+^ channels in a non-specific way. Ionomycin induces Ca^2+^ flux in BmMCs; it turned out that PDE3-deficient BmMCs showed a lower Ca^2+^ flux than control WT cells (**Supplementary Figure 3E**). Likewise, when WT BmMCs were pretreated with enoximone (20 µM), lower Ca^2+^ flux levels were reached (**Supplementary Figure 3F**). These data suggest that mast cell degranulation is, at least partly, regulated in a cAMP-Ca^2+^ dependent manner.

### PDE3 Inhibition Inhibits Primary Human Mast Cell Degranulation

**Figure 5:**
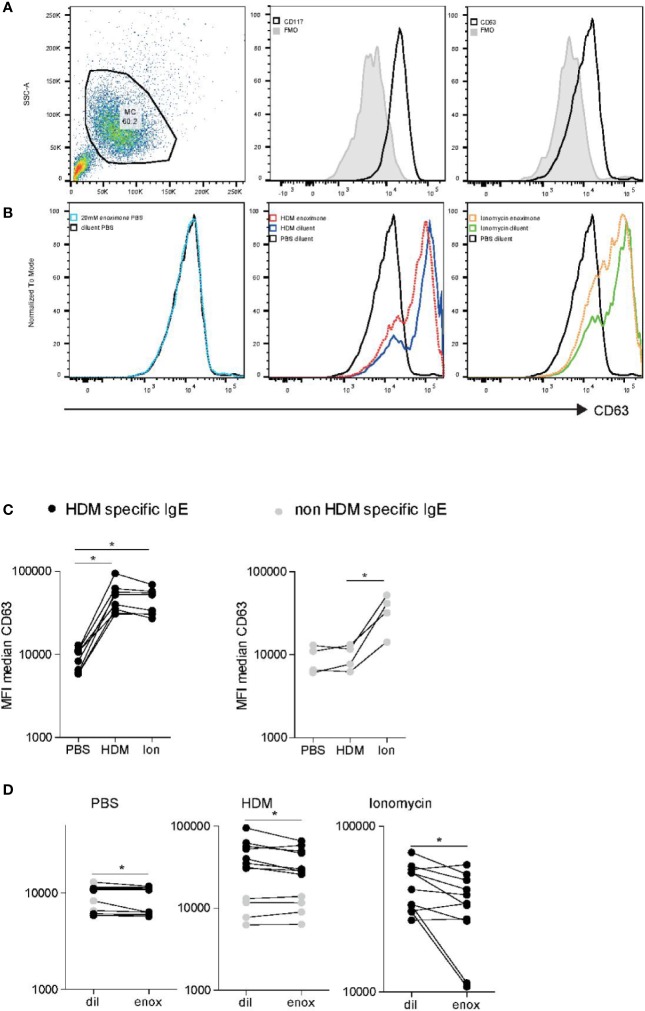
FcεR-mediated activation of human mast cell (HuMC) is impaired when PDE3 is targeted. **(A)** Flow cytometry analysis of cultured HuMC, gated on the basis of forward and side scatter characteristics. Histograms overlays show flow cytometry (FACS) plot and dot plots graphs illustrating the expression of CD117 and CD63, as indicated. **(B)** CD63 expression of enoximone (20 mM) pretreated passive sensitized HuMCs PBs control, HDM activated or ionomycin activated. Quantification of HDM-specific serum immunoglobulin E (IgE) [**(C)** left] or non-HDM specific IgE [**(C)** right]. CD63 expression of HuMCs treated with enoximone 20 µM and subsequently PBS stimulated [**(D)** left panel]. CD63 expression of HuMCs treated with enoximone 20 µM and subsequently HDM stimulated [**(D)** middle panel] or ionomycine stimulated [**(D)** right panel]. A Wilcox signed test was used; *P < 0.05. Data are shown from one out of two independent experiments. MFI, median fluorescence intensity; HDM, house dust mite.

To translate our findings to a more physiologically relevant model, we investigated the effects of PDE3 inhibition in primary human MCs. Peripheral blood-derived primary human MCs (HuMCs) were subjected to flow cytometric analysis (**Figure 5A**), notwithstanding the relatively high autofluorescence (**Figure 5A** left panel), CD117 (**Figure 5A** middle panel), and CD63 (**Figure 5A** right panel). Overnight passive sensitized (20% serum) HuMCs, with sera obtained from HDM mono-allergic or grass pollen (GP) mono-allergic donors were subjected to flow cytometric analysis (**Figure 5B**) after having treated with enoximone or diluent and stimulated with PBS (**Figure 5B** left panel), HDM (**Figure 5B** middle panel), or Ionomycine (**Figure 5B** right panel). The CD63 expression levels of HuMCs changed when the cells were passively sensitized with serum obtained from HDM-allergic donors (**Figure 5C** left panel) but not GP mono-allergic donors (**Figure 5C** right panel), followed by HDM stimulation (60 ng/ml), illustrating that only specific IgE cross-linking induces MC degranulation. Quantification indicated that HuMCs can, after passive sensitization, be used to measure specific FcεR cross-linking-dependent MC degranulation and ionomycine-dependent MC degranulation. CD63 expression was reduced when HuMCs were treated with enoximone 20 µM and subsequently PBS stimulated (**Figure 5D** left panel). MC degranulation induced by HDM (**Figure 5D** middle panel) or by ionomycine (**Figure 5D** right panel) was reduced by pretreatment with enoximone 20 µM. Recapitulating: both IgE-dependent and ionomycin (Ca^2+^)-dependent HuMC degranulation can be reduced by PDE3 inhibition.

## Discussion

This paper describe that MCs lacking PDE3, as well as LAD2 cells, HuMCs, and human basophils being treated with PDE3i, showed restrained degranulation. High intracellular levels of cAMP are associated with inhibition of MCs and basophil degranulation (Alm, 1984). Transgenic mouse MCs lacking PDE3 demonstrated that MCs without PDE3 react the same as those treated with PDE3i. We used clinically relevant PDE3i to investigate human basophils and human MCs illustrating the role of PDE3 in the degranulation of these cells in allergic airway inflammation.

MC degranulation is a process involving PDE3 and cAMP-PKA. Having investigated three different types of cells in addition to mouse MCs that lack PDE3 activity, all tests confirm the pathophysiological role of PDE3 in MCs/basophils. Our data show that inhibitors of cAMP-degrading enzymes (PDE3) or increased intracellular levels of dibutyryl cAMP (db-cAMP) prevent MC degranulation; this is consistent with the observation by Serra-Pages et al., showing that, when prostaglandin E_2_ (EP2)-dependent activation induces cAMP, cAMP/PKA suppresses IgE-/antigen-dependent signals and calcium influx, which is essential for MC degranulation (Serra-Pages et al., 2012). Shichijo et al. observed that cAMP-elevating agents, including PDE3i, inhibit cytokine production of MCs. Unfortunately, the concentrations used (up to 10 µM) were relatively low and were unable to reduce chemical mast cell mediators (Shichijo et al., 1999). PDE inhibition elevated cAMP and prevented MC activation (Shichijo et al., 1999). Measuring CD63 expression or the production of IL1b are accepted methods to determine SP-induced MC activation (Cop et al., 2018; Taracanova et al., 2018). Mast cell activation was measured in relation to CD63 expression. Cell surface CD63 is a marker associated with membranes of intracellular vesicles which is frequently used in basophil activation tests. In the current study cells were treated for 30–60 min with PDEi and subsequently stimulated as well for 30–60 min, which is longer than in previous studies that failed to see any PDE3-inhibitor effect. In addition, dose optimization appeared essential for every specific PDEi in order to find the best effective inhibitory dose in the bioassay. In general 10 µM is taken as a standard concentration for PDEi; however, this study illustrates that 10 µM is not always sufficient. We noticed that high concentrations (1 mM) of enoximone did not affect the cell viability. Sole enoximone treatment without stimulation had no effect on the expression of CD63 and CD203c in LAD2 cells. The initial effect of PDE3 inhibition is smooth muscle relaxation in humans. This effect is already noticeable within 30 s after IV treatment (Beute, 2014; Sobhy et al., 2019). In smooth muscle cells, increased cAMP inhibits myosin light chain kinase (MLCK), preventing Ca^2+^ binding, thereby reducing MLC phosphorylation, and hence relaxing the vascular smooth muscle (Prakash, 2016). This mechanism is not unique for smooth muscle cells (Prakash, 2016) or endothelial cells (Rigor et al., 2013) and could also offer an explanation on how, in a cAMP-dependent manner, MCs can be prevented to degranulate, with a role for calmodulin. PDE3 seems to be involved in truncation of FcϵRIβ and mediates Ca^2+^-dependent microtubule formation (Cruse et al., 2013), which prevents degranulation and cytokine release. The data in this paper indicate that the release of Ca^2+^ *via* low concentrations of ionomycin can be in part abrogated by PDE3 inhibition. This suggests that cAMP directly prevents binding of Ca^2+^, which in turn prevents microtubule formation and MC degranulation.

The reorganization of the actin cytoskeleton is under the spatial control of Ca^2+^ cAMP–PKA signaling in smooth muscle cells (Hocking et al., 2013). PDE3 plays a pathophysiological role in allergic airway inflammation in a translational model (Beute et al., 2018). The present study shows that targeting PDE3 has an inhibitory effect on exocytosis of MCs and can be seen as a valid bonus treatment in allergic asthma-related disease. PDE3 inhibitors are known to induce, within a few seconds, filamentous (F)-actin changes of the cytoskeleton in epithelial and endothelial cells (Liu et al., 2012), leading to enhanced barrier function. Calcium is important for MC degranulation and crucial for the granule migration in the cells, which is in counterbalance with cAMP (Draber et al., 2011). Ionomycin opens Ca^2+^ channels, probably *via* the microtubules and or actin cytoskeleton, leading to MC degranulation and activation. That this process can be inhibited in MCs by PDE3 inhibition is novel; how exactly this inhibition occurs is yet unknown.

Microdomains containing PDE3, PDE4, and adenylate cyclase (AC) balance each other out; using either PDE3- or PDE4-inhibitors, the net outcome might be in favor of immune attenuation. Others suggest that PDE activity is sufficient to restrict cAMP diffusion to specifically defined subcellular compartments in certain cell types (Terrin et al., 2006; Oliveira et al., 2010). These microdomains are formed by cytoskeleton and might constitute an efficient barrier to cAMP diffusion, and promote compartmentalized cAMP and PKA signals by allowing cAMP to accumulate locally to a level sufficient to activate PKA. This would prevent activation of PKA in the other locations of the cytosol (Rich et al., 2000). The actin cytoskeleton would act as a diffusional barrier for cAMP, and PDEs would be in control of local cAMP levels in thus created microdomains (Monterisi et al., 2012). Increased intracellular cAMP levels might account for the decreased reactivity of basophils to venom allergen (Bauer et al., 2007).

An important technique to illuminate this black box might be measuring phosphorylation of cell signaling molecules such as Syk Lyn, PLCγ, and PIP_2_, since PIP_2_ *via* activated PLCγ leads to IP_3_ and DAG. IP3 stimulates the IP_3_R on the ER, leading to the release of Ca^2+^. IP3R are phosphorylated by cAMP-dependent protein kinase (PKA) (Taylor, 2017).

The anti-inflammatory effects of PDE3 inhibitors have been established in other studies, and are illustrated by reduced serum TNFα levels during PDE3 inhibition, when compared to dobutamine-treated septic patients (Kern et al., 2001) and reduction of macrophages as well as CD11b expression on eosinophils (Beute et al., 2018). In alveolar epithelial cells LPS-induced biosynthesis of pro-inflammatory cytokines is regulated by cAMP and tightly controlled by PDEs, and can be reduced by PDE inhibitors (Haddad et al., 2002).

Allergen-driven degranulation of mast cells and basophils is one of the first steps in the allergy cascade resulting in local inflammation. In the context of allergic airway inflammation therapy the first choice to relieve symptoms is targeting basophil and/or MC activation, or blocking the effect of mast cell mediators. PDE4 inhibitors like roflumilast have shown to be beneficial in the context of allergic inflammation; however, because of the serious side effects they are not available for allergy related diseases (Gupta, 2012). PDE3 inhibitors are accompanied with less side effects (Metra et al., 2009) and are in use as therapeutics for intermittent claudication (Barnett et al., 2004). Clinical observations indicate immediate bronchodilation in asthma patients and animal models when treated with PDE3 inhibitors enoximone and milrinone (Howell et al., 1995; Fujimura et al., 1997; Myou et al., 1999).

Enoximone and its metabolites have the advantage over other PDE3/PDE4 inhibitors in that they have been clinically used for over 20 years, peri-operatively and in the ICU, to prevent organ failure, as well as in having minimal side effects. Using low doses of a clinically available PDE3 inhibitor like enoximone makes it possible to avoid the serious side effects that were often seen when asthma patients were treated with the very potent PDE4 inhibitors (Gupta, 2012). Investigational use of the PDE3 inhibitor enoximone shows improvement as well as attenuated cell-driven symptomatology in asthmatic and allergic patients (Beute unpublished). Clinical trials are underway to confirm these observation in asthma patients. PDE3 inhibition is not only a smooth muscle relaxant being beneficial in obstructive lung disease (Leeman et al., 1987), but also improve mucosal barrier function (Kern et al., 2001) and is in use as a rescue medication in severe asthma (Leeman et al., 1987; Kern et al., 2001; Beute, 2014; Sobhy et al., 2019). In conclusion, the present work has established that the small molecule PDE3 inhibitor enoximone is important in preventing basophil and mast cell degranulation. PDE3 is a clinically relevant therapeutic target in the context of allergic inflammation.

## Methods

### Animals

*Pde3a*^−/−^ and *pde3b*^−/−^ C57bl/6 mice were previously described (Masciarelli et al., 2004; Choi et al., 2006) and showed no abnormalities regarding T-cell development (data not shown). WT C57bl/6 mice were bought from Charles River. Animals were kept under specific pathogen-free conditions, provided with water and food *ad libitum* and were used at the age of 6–12 weeks.

### Cell Lines and Serum

We took LAD2 cells, kindly provided by Kirshenbaum and Metcalfe (National Institute of Allergy and Infectious Diseases, Bethesda, Maryland), and HMC1 cells, a gift from Dr. J.H. Butterfield (Mayo Clinic, Rochester, MN, USA) as a surrogate for MCs. The human MC line HMC1 was kindly provided by Dr. J.H. Butterfield (Mayo Clinic, Rochester, MN, USA). LAD2 is a stem-cell factor (SCF) dependent human MC line (kindly provided by Kirshenbaum and Metcalfe, National Institute of Allergy and Infectious Diseases, Bethesda, Maryland). Serum obtained from mono-allergic patients containing specific IgE for either house dust mite (62 - > 100 kU/l) or GrassPollen (68 kU/l) or (Birch >100 kU/l) was used for passive sensitization.

### Study Approval

Peripheral blood cells were isolated from buffy coats of blood donors (Sanquin Bloedvoorziening, NVT nr. 0014.03, Rotterdam, the Netherlands). Serum samples obtained from HDM and GP mono-allergic patients were initially collected during routine diagnostic analysis in the Laboratory Medical Immunology (Department of Immunology) of the Erasmus MC, Rotterdam, Netherlands, and remaining material was made available for this study. The Erasmus MC Medical Ethics Committee approved this availability without the need for informed consent, based on the use of fully anonymized remaining diagnostic material without the patient being subjected to additional risk or medical procedures. All animal experiments were approved by the Erasmus MC's Animal Ethics Committee.

### House Dust Mite Allergic Airway Inflammation Model

To induce chronic airway inflammation, mice were anaesthetized with isoflurane and treated intratracheally (i.t.) with 25 μg house dust mite (HDM; Greer Laboratories) allergen extract in 50 µl phosphate buffered saline (PBS) on day 0 or using PBS only (KleinJan et al., 2014; Vroman et al., 2017) three times per week, for six consecutive weeks; see **Supplementary Figure 2**.

### Therapeutic Intervention by Intratracheal Topical Application of Enoximone

Optimal dose of 25 µg enoximone *versus* diluent (Beute et al., 2018) was administered with 25 µg HDM solution and used for intratracheal treatment, or diluent (10% ethanol + 40% propylene glycol + 50% Milli-Q pH 12) was administered with 25 µg HDM solution. Controls were treated with enoximone 25 µg administered with PBS or diluent administered with PBS. Mice underwent enoximone treatment during the last 2 weeks of the challenge period; see **Supplementary Figure 1**.

### Histochemical Detection of Mast Cells

Toluidine blue, an aniline dye, stains mast cells metachromatically. Snap frozen lung tissue cryosections (6 µm) were histochemically stained with toluidine blue (0.5% in HCL 0.5 M) at pH 0.5 for at least 5 min and observed and quantified immediately (KleinJan et al., 1996). Quantification was performed by counting the number of toluidine blue positive cells per mm length basement membrane in the epithelium of bronchioles of the airways, using an Axioskop 20 microscope (Zeiss, Jena Germany) with an eyepiece graticule at a magnification of 200 x (Beute et al., 2018)

### Mouse Bone Marrow-Derived Mast Cells

Bone marrow (BM) cells were isolated from tibias and femurs of WT and KO mice. The bones were crushed, cells were filtered (100 µm) and washed. Red blood cells were lysed with lysis buffer. The cells were cultured in RPMI 1640 medium (Life Technologies) supplemented with 10 µg/ml interleukin 3 (IL-3), 10% heat inactivated fetal calf serum, 50 µg/ml gentamycin, 5*10-5 M β-mercaptoethanol at 37°C in 5% CO2 for 4–6 weeks. By week 4, 95–98% of the cells must be MCs (Cop et al., 2018). To confirm, the morphology of the bone marrow-derived MCs (BMMCs) was assessed by toluidine blue staining to observe mast cell granules.

Overnight DNP-specific IgE-sensitized MCs and PBS control MCs were stimulated with DNP-7 OVA 10 ng/ml for 30 min. Stained with antibodies against CD107b^AF647^ (Serotec) and CD200r^PE^ (eBiosciences) for 1 h at 4°C and washed. 4′,6-Diamidino-2-phenylindole (DAPI) was used as a live/dead marker. At least 2 * 10^3^ cells were used per condition.

### Analysis of Phosphodiesterase Expression

RNA was isolated from mouse bone marrow-derived MCs and LAD2 cells using a Total RNA Purification Kit (Sigma Aldrich). Purity of the extracted RNA was verified using a Nanodrop 2000 Spectrophotometer (Thermo Fisher Scientific). From the RNA, complementary DNA (cDNA) was reverse transcribed using a Revert Aid First Strand cDNA Synthesis Kit (Thermo Fisher Scientific). Quantitative PCR (qPCR) primers (for detailed description of primer sequences see (Liu et al., 2012) specific for all PDE3 and -4 isoforms were designed using the universal probe library (Roche Diagnostics), and checked for efficiency. Using SYBR Select Master Mix (Applied Biosystems), the cDNA was analyzed *via* qPCR, using a 7300 Real-Time PCR System (Applied Biosystems). Resulting data were normalized to relative expression of household genes [β-actin in human and hypoxanthine-guanine phosphoribosyl transferase (HPRT) in mouse]. Resulting data were further visualized using the comparative Ct (2^−ΔCt^) method (Wong and Medrano, 2005).

### Phosphodiesterases Inhibition of Basophils, LAD2 Cells, and Human Mast Cells

#### Basophils

PBMCs were isolated from buffy coats of healthy blood donors by lysing the red blood cells with lysis buffer (NH_4_CL) and washed with RPMI twice (Beute et al., 2018). Whole-blood cells were incubated with various PDE3 inhibitors (enoximone (10 µM), milrinone (100 µM), cilostazol (100 µM), diluent [10% ethanol + 40% propylene glycol + 50% Milli-Q and pH12) (Dil enox) or PBS or dimethyl sulfoxide (DMSO) resp.] for 30 min at 37°C next stimulated with anti-IgE 1 μg/ml and washed and stained with anti-CD63^PeCy7^, (eBioscience), anti-CD203c^PE^ (Coulter), anti-CD123^BV650^ (BD), anti-HLA-DR^BV711^ (BD), anti-FcϵRI^Fitc^ (eBioscience), anti-FcϵRI^APC^ (eBioscience), and anti-CD11c^AF700^(BD). DAPI or Aqua Live/Dead was used as live/dead marker. Basophils were gated as FcϵRI^+^CD123^+^CD11c^−^HLA DR^−^ cells. At least 5*10^5^ PBMCs were analyzed and a minimum of 1*10^3^ basophils were per condition were evaluated.

#### LAD2 Cells

LAD2 cells were sensitized passively with 20% serum overnight (serum was harvested from HDM sensitized donors (62 -> 100 kU/l), followed by washing steps to remove excess unbound serum-specific IgE. Then, the cells were treated with PDE3 inhibitor enoximone (10 or 50 µM) or diluent (10% ethanol + 40% propylene glycol + 50% Milli-Q and pH12) (Dil enox) for 30 min at 37°C, followed by stimulation with HDM [60 ng/ml (Greer Laboratories)] or 1 μM substance P (Sigma) for 1 h. Finally, the cells were washed followed by flow cytometry (FACS) staining with CD63 PeCy7 (eBioscience), CD203cPE (Coulter) for 1 h at 4°C and washed. DAPI was used as a live/dead marker. At least 2 * 10^3^ cells were used per condition.

#### Human Mast Cells

HuMCs were cultured as previous described (Gauvreau et al., 2000; Gaudenzio et al., 2016; Folkerts et al., 2020a; Folkerts et al., 2020b). In brief, CD34 progenitors were isolated according to the manufacturer's instructions in StemSpan medium (Stem Cell Technologies), supplemented with ciprofloxacin. Human CD34 progenitors were cultured in StemPro-34 medium, supplemented with 2 mM L-glutamine, 100 IU/ml penicillin, 100 g/ml streptomycin, 100 ng/ml recombinant human SCF (rhSCF), 100 ng/ml recombinant human interleukin (rhIL)-6, and 30 ng/ml rhIL-3 (first week only). Half of the cell culture medium was replaced weekly with media containing 100 ng/ml rhSCF and 100 ng/ml rhIL-6. Cells were used for experiments after 7 to 10 weeks in culture (Folkerts et al., 2020a; Folkerts et al., 2020b). CHO cells conditioned medium as a source of SCF was used from 3 months onwards. Overnight, passively sensitized HuMCs with 20% serum obtained from house dust mite (62 - > 100 kU/l) or grass pollen [68 kU/l), or birch pollen (> 100 kU/l)] sensitized mono-allergic donors were washed, three times, incubated with PDE3i enoximone 20 µM (1:1,000 20 mM) or diluent (10% ethanol + 40% propylene glycol + 50% Milli-Q and pH12) (Dil enox 1:1,000) for 60 min at 37°C. Subsequently, they were stimulated with HDM (60 ng/ml), ionomycine 0.5 µg/ml (Sigma), or diluent for 1 h at 37°C, washed, and stained with anti-CD63^PeCy7^ and anti-CD117 ^BV471^. At least 10*10^3^ cells were evaluated per condition.

### Statistics

Reported values are shown as mean ± SEM. Statistical analyses were performed with SPSS (SPSS Inc., Chicago, IL) using Kruskal–Wallis one-way ANOVA, followed by Mann-Whitney U-test. A Mann-Whitney U-test was only performed when the one-way ANOVA test pointed to significance. A Wilcoxon sign-rank test was used for paired samples. Resulting p values <0.05 were considered significant. Tests that did not reach significance (p > 0.05) are not indicated.

## Data Availability Statement

All datasets generated for this study are included in the article/**Supplementary Material**.

## Ethics Statement

The Erasmus MC Medical Ethics Committee approved this availability without the need for informed consent, based on the use of fully anonymized remaining diagnostic material without the patient being subjected to additional risk or medical procedures. All animal experiments were approved by the Erasmus MCs Animal Ethics Committee.

## Author Contributions

AK and JB contributed to study design and wrote and edited the manuscript. KG, HN, RH, VB, AK and JF contributed to performing the studies and obtaining the samples analyzed in this study. MS, SH, and RWH were involved in reviewing and shaping the manuscript.

## Conflict of Interest

JB has a patent PCT/NL2015/050246, P6054704PCT pending and is shareholder and owner of BMR b.v. Relatives of AK are shareholders of BMR b.v.

The remaining authors declare that the research was conducted in the absence of any commercial or financial relationships that could be construed as a potential conflict of interest.

## Supplementary Material

Supplementary Figure 1 PDE3 and PDE4 are expressed in mice mast cells. Expression of PDE3 and PDE4 isoforms in bone marrow-derived mice mast cells.

Supplementary Figure 2 Experimental design.Experimental chronic HDM-driven asthma design, showing intratracheal exposure to 25 μg of HDM or PBS that was performed three times per week, as indicated by arrows. From 5 weeks onwards HDM/saline was admixed with placebo (diluent) or 25 μg of enoximone. Analyses were performed at day 41, one day after the last challenge.

Supplementary Figure 3 Dose-response of cAMP and cGMP analogs on CD63 expression by SP-activated LAD2 cells. **(A-D)** CD63 expression of LAD2 cells pre-treated for 30 min with increasing doses of enoximone **(A)**, a cAMP analog **(B)**, a cGMP analog **(C)** and the PKA inhibitor (Rp)-8-Br-cAMP **(D)**, followed by stimulation with 1 μM substance P (SP) for 30 min. Data are shown as mean values ± SEM. A Mann-Whitney U test was used; * P < 0.05. **(E,F)** Ca2+ flux experiment with bmMCs obtained from *Pde3^-/-^* mice and WT **(E)** and from WT treated with PDE3i enoximone (20µM) or diluent **(F)**. Data are shown of one representative experiment from three independent experiments.

## Abbreviations

AAI, allergic airway inflammation; AHR, airway hyper-responsiveness; BAL, broncho-alveolar lavage; cAMP, cyclic adenosine monophosphate; cGMP, cyclic guanosine monophosphate; DC, dendritic cell; HDM, house dust mite; HRP, horseradish peroxidase; MLN, mediastinal lymph node; MC, mast cell; PBS, phosphate buffered saline; PDE, phosphodiesterase; PDE3i, phosphodiesterase 3 inhibitor(s)/inhibition; SP, substance P.
