# Supplementary figures and images for: PDE3 Inhibition Reduces Epithelial Mast Cell Numbers in Allergic Airway Inflammation and Attenuates Degranulation of Basophils and Mast Cells
Source: Front Pharmacol. 2020 May 1;11:470. doi: 10.3389/fphar.2020.00470 (PMC7206980; doi:10.3389/fphar.2020.00470)

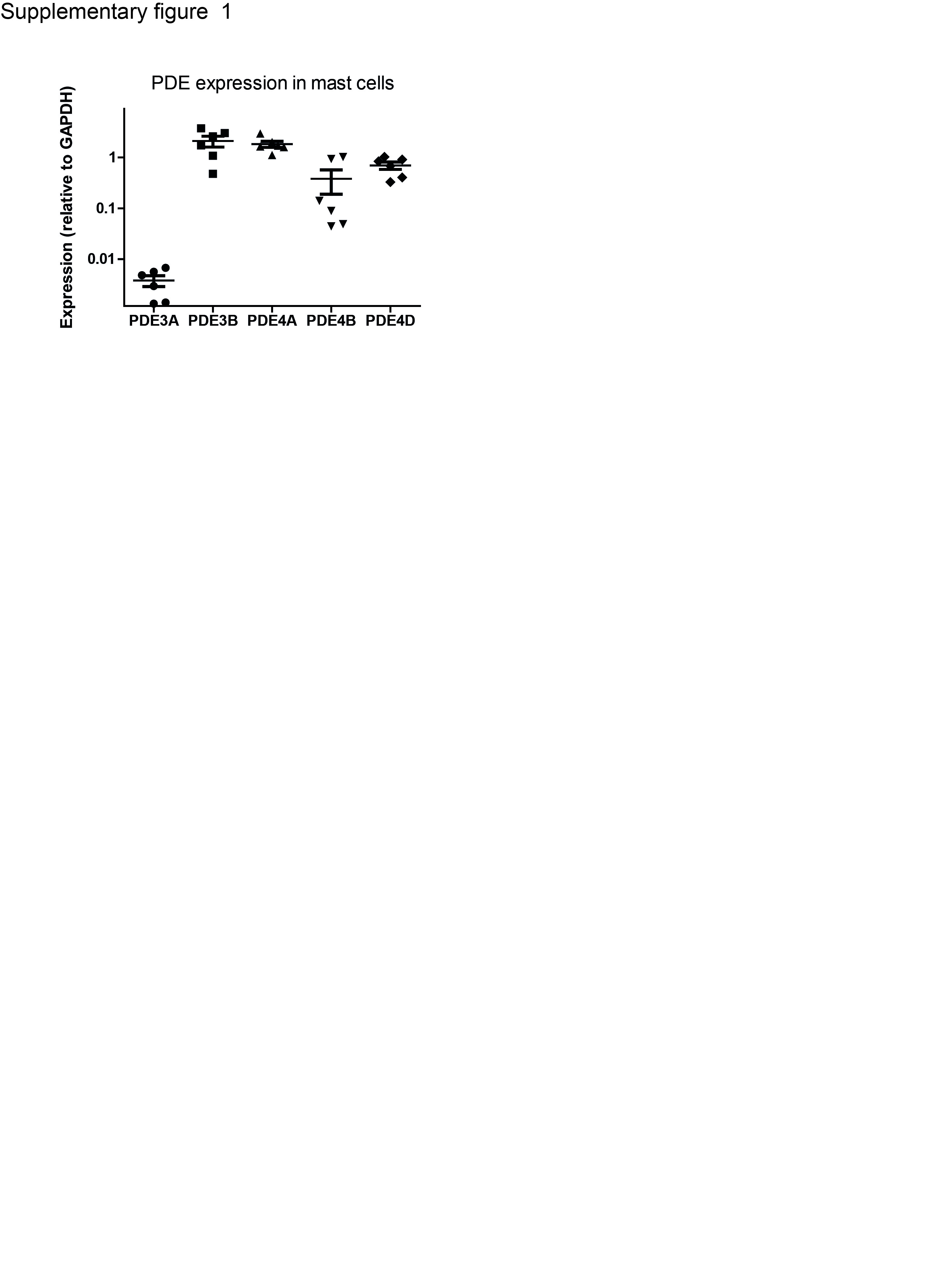

Supplement: Supplementary Figure 1 — PDE3 and PDE4 are expressed in mice mast cells. Expression of PDE3 and PDE4 isoforms in bone marrow-derived mice mast cells. [file Image_1.jpeg]

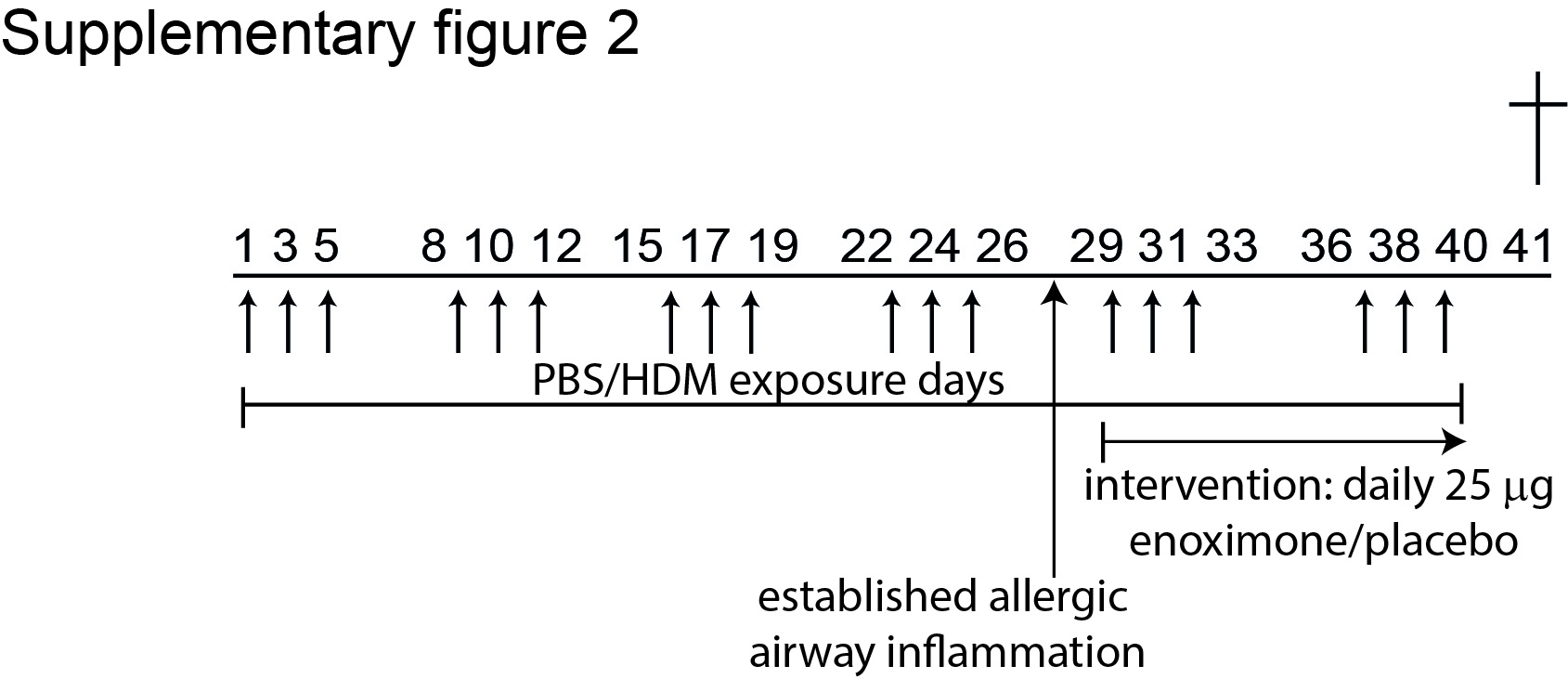

Supplement: Supplementary Figure 2 — Experimental design.Experimental chronic HDM-driven asthma design, showing intratracheal exposure to 25 μg of HDM or PBS that was performed three times per week, as indicated by arrows. From 5 weeks onwards HDM/saline was admixed with placebo (diluent) or 25 μg of enoximone. Analyses were performed at day 41, one day after the last challenge. [file Image_2.jpeg]

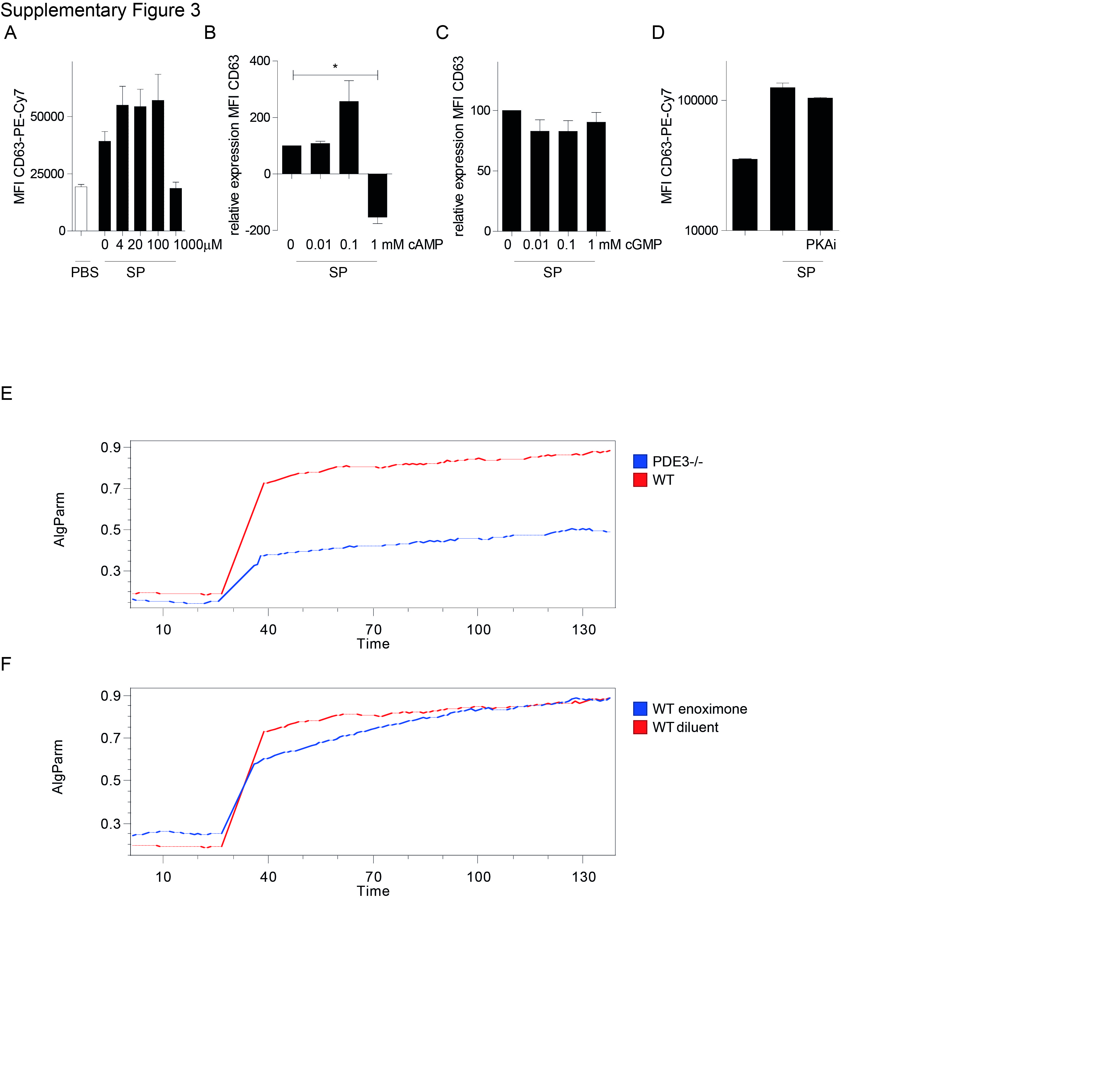

Supplement: Supplementary Figure 3 — Dose-response of cAMP and cGMP analogs on CD63 expression by SP-activated LAD2 cells. (A-D) CD63 expression of LAD2 cells pre-treated for 30 min with increasing doses of enoximone (A), a cAMP analog (B), a cGMP analog (C) and the PKA inhibitor (Rp)-8-Br-cAMP (D), followed by stimulation with 1 μM substance P (SP) for 30 min. Data are shown as mean values ± SEM. A Mann-Whitney U test was used; * P < 0.05. (E,F) Ca2+ flux experiment with bmMCs obtained from Pde3-/- mice and WT (E) and from WT treated with PDE3i enoximone (20µM) or diluent (F). Data are shown of one representative experiment from three independent experiments. [file Image_3.jpeg]
